# The role of spatial heterogeneity in the evolution of local and global infections of viruses

**DOI:** 10.1371/journal.pcbi.1005952

**Published:** 2018-01-25

**Authors:** Koich Saeki, Akira Sasaki

**Affiliations:** 1 Department of Evolutionary Studies of Biosystems, SOKENDAI (The Graduate University for Advanced Studies), Hayama, Kanagawa, Japan; 2 Evolution and Ecology Program, International Institute for Applied Systems Analysis, Laxenburg, Austria; University of California Irvine, UNITED STATES

## Abstract

Viruses have two modes spread in a host body, one is to release infectious particles from infected cells (global infection) and the other is to infect directly from an infected cell to an adjacent cell (local infection). Since the mode of spread affects the evolution of life history traits, such as virulence, it is important to reveal what level of global and local infection is selected. Previous studies of the evolution of global and local infection have paid little attention to its dependency on the measures of spatial configuration. Here we show the evolutionarily stable proportion of global and local infection, and how it depends on the distribution of target cells. Using an epidemic model on a regular lattice, we consider the infection dynamics by pair approximation and check the evolutionarily stable strategy. We also conduct the Monte-Carlo simulation to observe evolutionary dynamics. We show that a higher local infection is selected as target cells become clustered. Surprisingly, the selected strategy depends not only on the degree of clustering but also the abundance of target cells per se.

## Author summary

Viruses such as human immunodeficiency virus and measles virus can spread through physical contact between infected and susceptible cells (cell-to-cell infection), as well as normal cell-free infection through virions. Some experimental evidences support the possibility that high ability of cell-to-cell infection is selected in the host. Since the mode of spread affects the evolution of life history traits, it is important to reveal what condition favors high ability of cell-to-cell infection. Here we address what level of cell-to-cell infection is selected in different target cell distributions. Analysis of ordinary differential equations that keep track of dynamics for spatial configuration of infected cells and the Monte-Carlo simulations show that higher proportion of local infection is selected as target cells become clustered. The selected strategy depends not only on the degree of clustering but also the abundance of target cells per se. Our results suggest viruses have more chances to evolve the ability of local infection in a host body than previously thought. In particular, this may explain the emergence of measles virus strains that gained the ability to infect the central nervous system.

## Introduction

Viruses have evolved various mechanisms to spread within a host body and between hosts. There are two modes for viral spread in a host body, one is to release infectious particles (virions) from the infected cells into the extracellular medium, and the other is to infect directly from an infected cell to an adjacent cell. The mode of viral spreading depends on the type of virus, their target cells and tissues. For example, viruses that lyse the host cell rely on the release of virions as the only way of spreading. In contrast, viruses that exit host cells by budding or some forms of exocytosis have a potential to spread directly from cell to cell. Conceptually the simplest mechanism of cell-to-cell spread is the fusion of infected and uninfected cells. To enter into a host cell, some viruses have proteins that cause membrane fusion, and these fusion proteins are expressed on the cell surface after viral replication is initiated. Thus, fusion proteins on the infected cell may cause membrane fusion to the adjacent uninfected cell, resulting in a single giant cell (syncytium). For instance, vaccinia virus forms two different forms specific to each mode: mature virus released after lysis of infected cells, and double membrane-enveloped extracellular virus that remains associated with the producer cell surface and spreads by cell-to-cell [1,2]. Influenza virus have the potential to spread in a cell-to-cell manner but inherently release virions [3,4]. Other more sophisticated mechanisms of cell-to-cell virus spread also exist (for more examples, see [5]).

Both cell-free and cell-to-cell modes of viral spread have their own advantages and disadvantages [5]. Since virions are much smaller and more resistant to environmental change than infected cells, they can disperse farther from the infected cell and even outside the infected host. However, cell-free infection takes a longer time for the virus to encounter a target cell and to engage attachment and entry receptors because the new infection event depends on diffusion and kinetic processes. This is particularly disadvantageous for viruses that bind to receptors that have low expression on host cells and/or those that must engage multiple receptors in order to enter the cell. Immunological barriers to free virions such as antibodies, complement, defensins and macrophages are also factors that discourage cell-free mode. In contrast, viruses that use cell-to-cell infection can avoid many of such obstacles. Another advantage of cell-to-cell infection is the efficiency of new infections: once an infection has occurred, the cell-to-cell mode of viral spread eliminates the rate-limiting step of diffusion. The disadvantage of cell-to-cell infection is a locality of new infection: as infections progress, "self-shading" occurs whereby host cells near infected cells decrease. Cell-mediated immunity, mainly caused by killer T cells, also has a large impact to cell-to-cell infection because infected cells are clustered.

The present study focuses on the intra-host evolution of cell-free and cell-to-cell infection, which we refer to as global and local infection respectively, with spatial structure of target cells. Since the mode of spread of a pathogen affects the evolution of its life history traits, such as virulence [6–12], it is important to reveal what level of global and local infection is selected. Previous studies assumed that there is a trade-off between global and local infection [6–12]. Our present study also uses this assumption that denotes the retention of virions on the infected cell surface and polarization to the side of cell-cell contact promote the efficient local infection but interfere with global infection [13]. Because virions could be transmitted to different susceptible cells during global infection, local infection can “waste” some virus particles by putting them all into one cells. As an example, previous study that addressed the evolution of global versus local spread, assuming spatial host dynamics whereby reproduction of host individuals is always local and, as a result, the host population is spatially structured [14]. They showed that the spatial structure is important for the evolution of local infection but this conclusion is limited to particular spatial structures generated by infection dynamics and host reproduction. Such self-organized structures are only a part of possible spatial structures; spatial distribution of target cells in a body, for example, have far more diversity than those self-organized by a particular epidemiological model. Another example of previous theoretical studies include local infection through virological synapses in retroviruses [15,16]. These studies considered viruses that spread via cell-free and cell-to-cell infection, and viral strategy is defined as the number of viruses passed per virological synapse. Since total number of virions produced before an infected cell dies is limited, putting virions all into one cell can “waste” those virus particles. The strategy selected in evolutionary competition can be an intermediate number of viruses passed per synapse (i.e. evolved viruses make use of local infection) depending on the viral kinetics. However the effect of spatial structure was only implicitly discussed. To date, the relationship between a measure of spatial structure and an evolutionary outcome remains unclear in the literature.

In the ecological context, our focus is interpreted as the evolution of short- vs. long-range dispersal. Harada [17] modeled population dynamics in a lattice-structured habitat and assumed a linear trade-off between global and local dispersal. In this model, the assumption that vacant sites are always available also causes the similar limitation as in Kamo and Boots [14]. Hiebeler [18,19] assumed the mixture of suitable and unsuitable habitats in the lattice space in which individuals reproduce globally and locally. In these works, pairwise invasibility was examined by Monte-Carlo simulation but the evolution of the proportion of global reproduction along an adaptive dynamics framework was not discussed.

The purpose of this paper is to analyze how the evolutionarily stable proportion of global (or local) infection is related to spatial heterogeneity. We model the evolution of the proportion of global and local infection in a spatially structured SIS model in which some sites are occupied by target cells and other sites are occupied by non-target cells on a lattice space. For simplicity, we assume that whether a site is occupied by a target cell or a non-target cell will never change. Although it may seem unrealistic to assume that an infected cell directly return to susceptible state (SIS model), this would be justified if a susceptible cell fills a blank immediately after an infected cell dies. The manner of target and non-target cell distribution over the lattice space was parameterized by the frequency of target cells and the pair frequency of target cells. We also assume viruses have an ability to establish persistent infection like human immunodeficiency viruses (HIV) that have enough time for within-host evolution and thus adaptive dynamics can be applied. The dependence of evolutionary outcomes on parameters that denote spatial structure is the main focus of our research, which has not been clearly shown in previous studies. At first, the infection dynamics are modeled by pair approximation and the endemic condition for a virus with a certain proportion of global infection is calculated. Next, the evolutionarily stable strategy is obtained by an adaptive dynamics framework. Finally, we conduct the Monte-Carlo simulation and compare results with analytical results.

### Model

#### Spatial structure of cells

We consider a two-dimensional lattice space in which each site is occupied by either a target cell or a non-target cell for a particular virus. The state of target cells dynamically change between susceptible (S) and infected (I) whereas non-target cells (O) never change.

Let *x*_*σ*_ be the global density of the sites with state *σ* in the population (*σ* is either S, I, or O), and *p*_*σσ*_, be the probability that a randomly chosen site has state *σ* and one of its randomly chosen nearest neighbors has state *σ*′. Throughout this paper, we call *p*_*σσ*_, pair density. The conditional probability that a randomly chosen *σ* site has a *σ*′ at its nearest neighbor is denoted by *q*_*σ*′/*σ*_ = *p*_*σσ*′_/*x*_*σ*_. By definition, we have a following relationship about *p*_*σσ*′_

$$p_{\sigma \sigma \prime }=x_{\sigma }q_{\sigma^{\prime }/\sigma }=x_{\sigma \prime }q_{\sigma /\sigma \prime }=p_{\sigma^{\prime }\sigma }.$$ (1)

Note that *x*_*σ*_ = ∑_σ′_*p*_*σσ*′_. We define two parameters to characterize the spatial configuration: *x*_*C*_, the global density of target cells, and *p*_*CC*_, the pair density of target cells. One of the main interests of this study is the dependence of the evolutionary outcome on *x*_*C*_ and *p*_*CC*_. In a pair of two neighboring target cells, three possible states should be considered (SS, SI, and II), where the density of IS state is exactly the same as SI state due to Eq (1). The pair density of two target cells in the population is fixed to *p*_*CC*_, and hence

$$p_{SS}+2p_{SI}+p_{II}=p_{CC}.$$ (2a)

On the other hand, there are two states (SO and IO) in a pair of target and non-target cells. The sum of these densities is constant and because *x*_*C*_ = *p*_*CC*_ + *p*_*CO*_,

$$p_{SO}+p_{IO}=p_{CO}=x_{C}-p_{CC}.$$ (2b)

**Fig 1:**
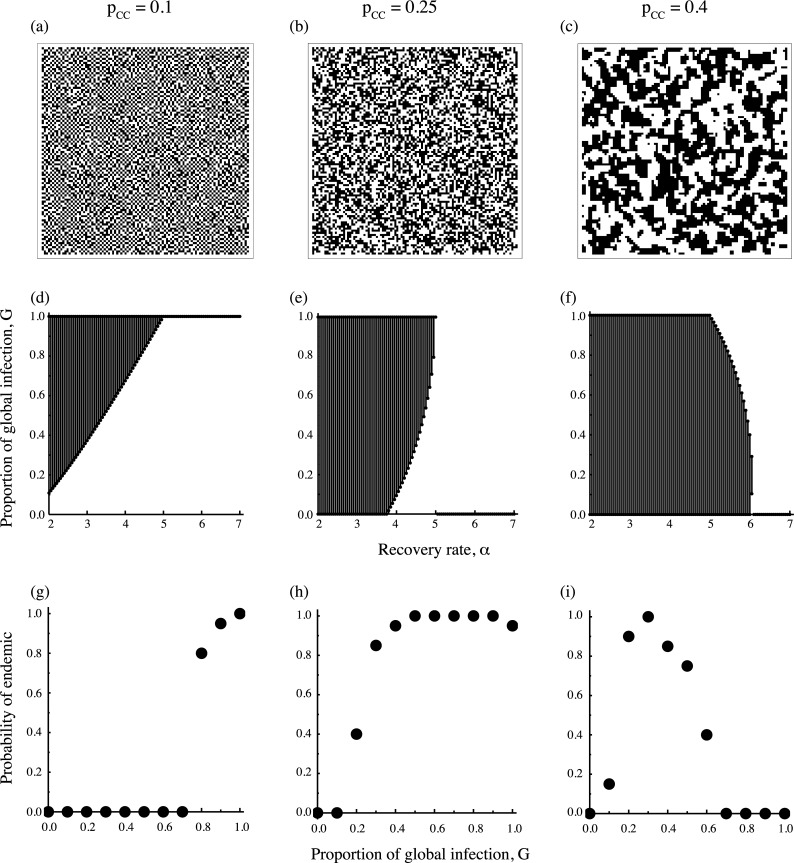
Epidemiological dynamics on heterogeneous spatial configurations of target cells randomly generated with varying pair correlation *p*_*CC*_ but fixed mean density of target cell *x*_*C*_ = 0.5. Each column denotes the configuration for *p*_*CC*_ = 0.1 (a, d, g), *p*_*CC*_ = 0.25 which corresponds to complete spatial randomness (b, e, h), and *p*_*CC*_ = 0.4 (c, f, i). (a-c) Examples of spatial structure, where target cells are shown in white. (d-f) The regions in the parameter space of recovery rate (*α*) and the proportion of global infection (*G*) in which viruses are either maintained in endemic equilibrium (shaded) or go extinct (white). The endemic condition is obtained by Eq (7). (g-i) The results of Monte-Carlo simulation showing the fraction of trials in 20 independent simulation runs in which viruses didn’t become extinct until 500 time steps as a function *G*. The recovery rate is fixed at *α* = 4 (g, h), or *α* = 5.5 (i). Other parameters are *β*_*G*_ = *β*_*L*_ = 10.

Although there are six possible states in a pair, only three of them are independent because we have three constants, the total fraction of sites (*x*_*S*_ + *x*_*I*_ + *x*_*O*_ = 1), the global density of target cells *x*_*C*_, and the pair density of target cells *p*_*CC*_. The degree of cell clustering is denoted by $p_{CC}/x_{C}^{2}$, where $p_{CC}/x_{C}^{2}=1$ is equivalent to zero spatial correlation and called complete spatial randomness (CSR). Target cells are aggregated more than CSR when $p_{CC}/x_{C}^{2}>1$, whereas cells are more uniform than CSR when $p_{CC}/x_{C}^{2}<1$ (See Fig 1A–1C).

#### Infection dynamics

The spatially structured infection dynamics is modeled by pair approximation and the main scheme is the same as Boots and Sasaki [8]. Let global and local infection rate be *β*_*G*_ and *β*_*L*_ respectively, and we define the proportion of global infection *G* (0 ≤ *G* ≤ 1) as a trait that viruses can change through mutation. Then, for a susceptible cell, the probability of getting infected by global infection per unit time is *β*_*G*_*Gx*_*I*_. In case of local infection, there are two possibilities: infection between cells that we are focusing on or infection from an infected cell standing at the other 3 neighboring sites. The probabilities of getting infected by local infection per unit time are *β*_*L*_(1−*G*)*θ* for the former case, and *β*_*L*_(1−*G*)(1−*θ*)*q*_*I/S*_ for the latter case, where *θ* is the inverse of the number of nearest neighbors for each site (*θ* = 1/4 for our two-dimensional lattice situation). For convenience, we use following expressions,

$$\phi =\beta_{G}Gx_{I}+\beta_{L}(1-G)(1-\theta )q_{I/S}=gx_{I}+l\frac{p_{SI}}{x_{S}},$$ (3a)

$$\psi =\beta_{L}(1-G)\theta ,$$ (3b)

where *g* = *β*_*G*_*G*, and *l* = *β*_*L*_(1−*G*)(1−*θ*). Using Eq (3), the probability that a susceptible cell in a SI pair gets infected per unit time interval is expressed as *ϕ* + *ψ*. Infected cells either die and are immediately filled by new susceptible cells, or recover to susceptible state with rate *α*. To express infection dynamics by pair approximation, we choose *x*_*I*_(≡ *p*_*SI*_ + *p*_*II*_ + *p*_*IO*_), *p*_*SI*_ and *p*_*IO*_ as three independent variables,

$$\frac{dx_{I}}{dt}=\left\{gx_{S}+(l+\psi )\frac{p_{SI}}{x_{I}}-\alpha \right\}x_{I},$$ (4a)

$$\frac{dp_{SI}}{dt}={-(\phi +\psi +\alpha )p}_{SI}+\phi p_{SS}+\alpha p_{II},$$ (4b)

$$\frac{dp_{IO}}{dt}={\phi p}_{SO}-\alpha p_{IO}.$$ (4c)

For more explanation, see equations (A1) and (A2) in S1 Text.

To consider the evolution of virus trait value *G*, we use invasion analysis which examines whether a mutant strain can increase or not when it arises at a resident equilibrium. We can obtain analytical expressions of the resident’s equilibrium densities for a given value of *G* from implicit relationship (A6)-(A8) in Appendix A. A resident equilibrium exists as long as the parameters satisfy the endemic condition that is obtained in the next section. At the resident equilibrium, mutants (denoted by subscript *J*) with proportion of global infection is *G*′, are introduced with a small initial frequency. In the full dynamics of residents (with *G*) and mutants (with *G*′), there are ten possible pair densities but we only have to consider seven pairs due to the three constraints, Eq (2) and *x*_*S*_ + *x*_*I*_ + *x*_*O*_ = 1. The dynamics are obtained by adding four more equations to the Eq (4):

$$\frac{dx_{I}}{dt}=\left\{gx_{S}+(l+\psi )\frac{p_{SI}}{x_{I}}-\alpha \right\}x_{I},$$ (5a)

$$\frac{dp_{SI}}{dt}={-(\phi +\psi +\alpha )p}_{SI}+\phi p_{SS}+\alpha (p_{II}+p_{IJ}),$$ (5b)

$$\frac{dp_{IO}}{dt}={\phi p}_{SO}-\alpha p_{IO}.$$ (5c)

$$\frac{dx_{J}}{dt}=\left\{g\prime x_{S}+(l\prime +\psi \prime )\frac{p_{SJ}}{x_{J}}-\alpha \right\}x_{J},$$ (5d)

$$\frac{dp_{SJ}}{dt}={-(\phi +\phi \prime +\psi \prime +\alpha )p}_{SJ}+\phi \prime p_{SS}+\alpha (p_{JJ}+p_{IJ}),$$ (5e)

$$\frac{dp_{IJ}}{dt}={\phi p}_{SJ}+\phi \prime p_{SI}-2\alpha p_{IJ},$$ (5f)

$$\frac{dp_{JJ}}{dt}=2\left\{(\phi \prime +\psi \prime )p_{SJ}-\alpha p_{JJ}\right\}.$$ (5g)

where *g*′, *l*′, and *ψ*′ are as defined before but the definition of *G* is replaced by *G*′, and *ϕ*′ = *g*′*x*_*J*_ + *l*′*p*_*SJ*_/*x*_*S*_,. From the dynamics of resident and mutant, the linearized dynamics of a mutant strain around the resident equilibrium are obtained. If the mutant can increase its density, which is judged by inequality (B9) in S1 Text, we infer that the mutant can invade the resident population. To find an ESS, we assume 101 different possible strains with equally divided *G* values from *G*_*min*_ = 0 to *G*_*max*_ = 1, and pairwise invasibilities are checked between two neighboring strains. If a strain prevents the invasion by adjacent strains, we regard it as a local ESS.

#### Monte-carlo simulation

To confirm these analytical predictions, we also conducted Monte-Carlo simulations in a 100 ×100 lattice space with periodic boundary conditions. At first, two parameters *x*_*C*_ and *p*_*CC*_ are determined. Next, according to the parameters, Metropolis-Hastings algorithm generates the spatial structure as follows: 1) each site is made to be occupied by a target cell with probability *x*_*C*_, 2) exchange the states of two randomly chosen sites if current *p*_*CC*_ approaches to the goal by exchanging, 3) repeat step 2 until current *p*_*CC*_ becomes sufficiently close to the goal. For infectious dynamics of a single strain, each target cell changes its state in a short time interval with some probability that is expected by ordinary differential equations Eq (4). In the simulation of evolutionary dynamics, we also assume 101 different possible strains and each strain can change by mutation to a strain of adjacent *G* value at a certain rate. Other infectious dynamics, occurrence of new infection and transition from infected to susceptible, is similar to the case of a single strain.

## Results

### Condition for endemic equilibrium

For the first step, we checked whether a strain with a certain *G* value can be endemic or not by using the stability analysis of the disease free equilibrium. A similar analysis is done by Hiebeler [18] but the endemic condition was calculated for extreme cases (*G* = 0 or *G* = 1) in that study. Here we showed that the endemic condition is also obtained for a virus strain with intermediate *G* value. In addition to the stability analysis, we also obtained the next generation matrix [20] from the linearized dynamics and calculated basic reproductive number (*R*_0_), as,

$$R_{0}=\frac{1}{2\alpha }\left(gx_{C}+lq_{C/C}+\sqrt{{\left(gx_{C}+lq_{C/C}\right)}^{2}+4g\psi p_{CC}}\right),$$ (6)

where *g* = *β*_*G*_*G*,*l* = *β*_*L*_(1−*G*)(1−*θ*), and *ψ* = *β*_*L*_(1−*G*)*θ*. The derivation is shown in Appendix A (S1 Text). When *G* = 1, *R*_0_ is *β*_*G*_*x*_*C*_/*α* ≡ *ρ*_1_, which is consistent with the result from the SIS model without spatial structure. In this case, the infection becomes endemic when *ρ*_1_ > 1 that means an infected cell infects more than one susceptible cell. When *G* = 0, *R*_0_ is *β*_*L*_(1−*θ*)*q*_*C/C*_/*α* ≡ *ρ*_0_. *ρ*_0_ > 1 is consistent with the "dyad heuristic" of Levin and Durrett [21], that is, a pair of infected cells will reproduce more than one pair of infected cells. For general values of *G*, the endemic condition is obtained by stability analysis of the disease free equilibrium,

$$\alpha^{2}-\alpha (gx_{C}+lq_{C/C})-g\psi p_{CC}<0.$$ (7)

By solving (7) with respect to *α*, the result becomes consistent with *R*_0_ > 1. For the intermediate value of *G*, the condition (7) is rewritten by using *ρ*_1_ and *ρ*_0_,

$$\left[1-\frac{1}{\rho_{1}G}\right]\left[1-\frac{1}{\rho_{0}(1-G)}\right]<\frac{1}{1-\theta }.$$

Fig 1 shows the region of *G* that satisfies endemic condition (7) with changing the recovery rate *α*. The difference among Fig 1D–1F is the degree of spatial correlation, $p_{CC}/x_{C}^{2}$ (but it is not exactly same as the spatial correlation $\left(p_{CC}-x_{C}^{2}\right)/\left(x_{C}-x_{C}^{2}\right)$). Of course, viruses cannot be endemic when the recovery rate *α* is too high. With increasing *α*, highly locally infecting strains drop out first when host cells distribute like CSR or when cells distribute more uniform than CSR. On the other hand, strains with intermediate *G* value are more resistant to the increase of *α* than other strains when host cells distribute with a positive correlation. Results of the simulation (Fig 1G–1I) indicate that the probability of survival in several trials show similar dependence as predicted by condition (7).

### Invasibility analysis and evolutionarily stable strategy

**Fig 2:**
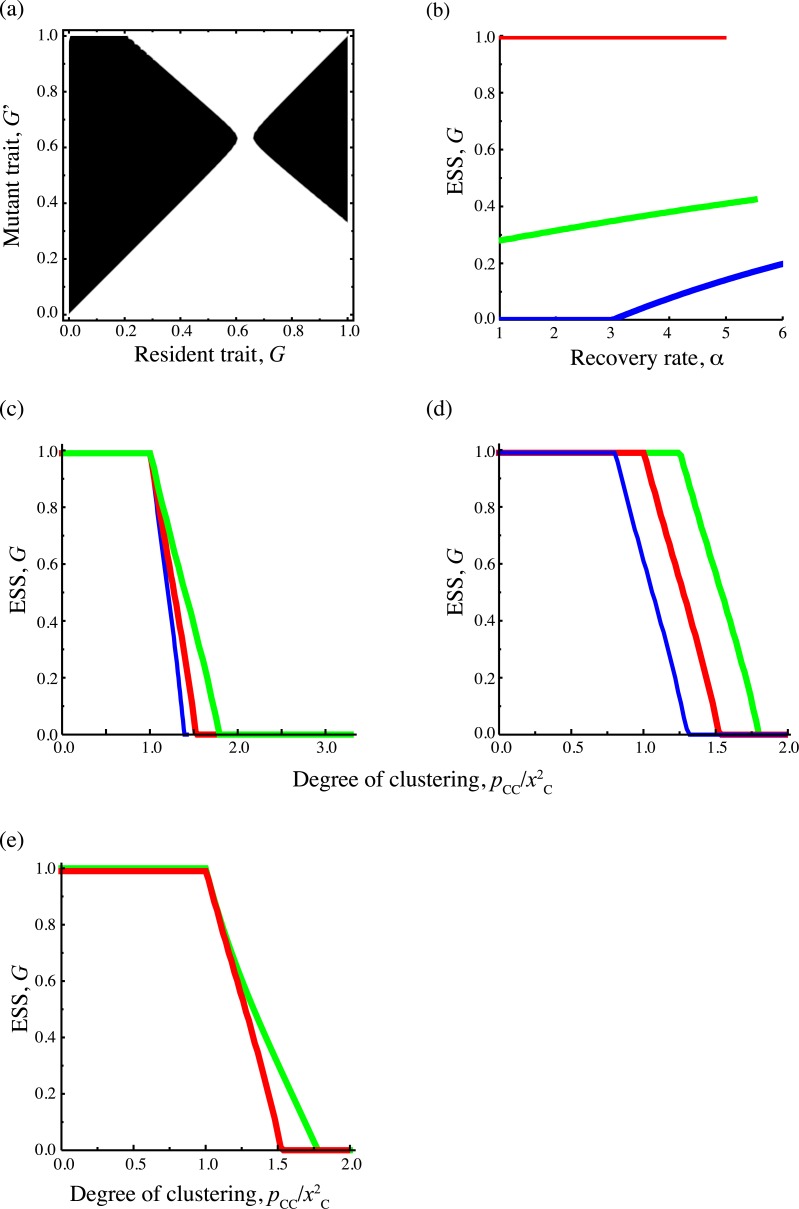
The results of pairwise invasibility analysis. (a) An example of pairwise invasibility plot with *x*_*C*_ = 0.5, *p*_*CC*_ = 0.3, *α* = 1, *β*_*G*_ = *β*_*L*_ = 10. The black region shows that a mutant strain can invade a resident population. (b)-(d) The parameter dependence of evolutionarily stable proportion of global infection. The parameters except denoted below are the same as (a). (b) Dependence on recovery rate *α*. The red line denotes the evolutionarily stable (ESS) proportion of global infection *G* for *p*_*CC*_ = 0.25, the green line denotes that for *p*_*CC*_ = 0.35, and the blue line denotes that for *p*_*CC*_ = 0.4. (c) Dependence of ESS *G* on the degree of clustering $p_{CC}/x_{C}^{2}$, which are altered by changing *p*_*CC*_ for a fixed *x*_*C*_. The red line denotes the results for *x*_*C*_ = 0.5, the green line for *x*_*C*_ = 0.4, and the blue line for *x*_*C*_ = 0.7. (d) Dependence of ESS *G* on the degree of clustering, which is changed as in (b). The red line denotes results for *β*_*G*_ = *β*_*L*_ = 10, the green line for *β*_*G*_ = 10, *β*_*L*_ = 8, and the blue line for *β*_*G*_ = 8, *β*_*L*_ = 10. (e) The red line denotes the ESS *G* and the green line denotes *G* values that maximize *R*_0_.

Using the invasibility analysis, we drew a pairwise invasibility plot (PIP); Fig 2A is an example of a single parameter set in which intermediate value of *G* is evolutionarily stable strategy (ESS). In all parameter region examined, there is a unique ESS and ESS strategy is any of completely global (*G* = 1), a mixture of local and global infections (an intermediate *G*), or completely local (*G* = 0). In the present model, there are no other patterns like the evolutionary branching, or more than two evolutionary singular points in the present model. The dependence of ESS on the parameters is shown in Fig 2B–2D. When target cells are relatively less clustered like CSR, ESS *G* is independent of *α* (red line in Fig 2B). In contrast, when target cells are relatively clustered, increase of *α* makes the ESS proportion of global infection higher (green and blue lines in Fig 2B). This is because high recovery rate increases the density of disease-free clusters, which makes global infection beneficial in accessing the isolated clusters. This dependence is also observed with *β*_*G*_ < *β*_*L*_ (S1 Fig). In Fig 2C and 2D, the dependence of ESS on the degree of clustering for target cells, *p*_*CC*_/*x*_*C*_^2^, is shown. In general, the higher the degree of target cell clustering becomes, the more local infection is optimal. When the rates of global and local infection are equal (*β*_*G*_ = *β*_*L*_), the threshold below which the completely global infection becomes ESS is CSR, $p_{CC}/x_{C}^{2}=1$ (Fig 2C). In addition to the dependence on *p*_*CC*_/*x*_*C*_^2^, the ESS proportion of global infection also depends on the fraction of cells *x*_*C*_ alone (Fig 2C). If *x*_*C*_ becomes higher with fixing $p_{CC}/x_{C}^{2}$, the ESS level of global infection becomes lower. The reason is that in this alteration, the conditional probability that a randomly chosen target cell has a target cell at its nearest neighbor, *q*_*C/C*_ = *p*_*CC*_/*x*_*C*_, becomes higher. Thus, local infection becomes more efficient in finding susceptible cells than global infection. The threshold point at which the completely global strain cannot be ESS does not change by altering *x*_*C*_, which is analytically shown in the next section. When the two infection rates differ, the ESS *G* value tends to prefer the infection mode of better efficiency (Fig 2D). It should be noted that the ESS proportion of global infection is not always a *R*_0_ maximizing strategy (Fig 2E). When $p_{CC}/x_{C}^{2}$ is very high, the ESS proportion is much lower than the *G* value that maximizes R_0_. In most epidemiological or infection dynamics without structure, the ESS trait is to maximize R_0_ [22,23]. When cells are spatially clustered, however, the ESS is not always maximizing R_0_, which may be due to a “self-shading” problem as discussed in a previous study [8].

### Invasivility to the completely global strain

Here we consider the special case in which a mutant strain with a certain *G*′ (< 1) invades a completely global resident strain (*G* = 1). The endemic equilibrium of the completely global strain is obtained from Eq (4) (for the calculation, see equations (A9) in S1 Text),

$${\hat{x}}_{I}=x_{C}-\frac{\alpha }{\beta_{G}},$$

$${\hat{p}}_{SI}=\left(1-\frac{\alpha }{\beta_{G}x_{C}}\right)\frac{\alpha }{\beta_{G}x_{C}}p_{CC},$$

$${\hat{p}}_{IO}=\left(1-\frac{\alpha }{\beta_{G}x_{C}}\right)(x_{C}-p_{CC}),$$

where ${\hat{x}}_{I},\;{\hat{p}}_{SI}$ and ${\hat{p}}_{IO}$ are pair densities at the equilibrium. In this case, we can analytically obtain the condition for a mutant strain to increase its density around the resident's endemic equilibrium (for derivation, see Appendix B in S1 Text),

$$\frac{\beta_{G}}{\beta_{L}(1-\theta +G^{\prime }\theta )}<\frac{p_{CC}}{x_{C}^{2}}.$$ (8)

The right-hand side of (8) denotes the degree of spatial correlation, and this is the reason why we choose $p_{CC}/x_{C}^{2}$ as the horizontal axis of Fig 2C and 2D. The left-hand side of (8) represents an increasing function of the mutant’s proportion of global infection *G’*. Therefore if $\beta_{G}/\beta_{L}>p_{CC}/x_{C}^{2}$ holds, the completely global strain prevents any kind of mutant from invading and it is an ESS. Especially when *β*_*G*_ = *β*_*L*_, the threshold of the spatial configuration at which the completely global strain can be the ESS corresponds to complete spatial randomness (CSR). It means that when cells distribute with a negative correlation, the completely global strain is the ESS, but when cells distribute with a positive correlation, some degree of contact infection can be beneficial. As predicted in this section, the threshold point moves to *β*_*G*_/*β*_*L*_ when *β*_*G*_ ≠ *β*_*L*_ (Fig 2D).

### Monte-Carlo simulation of evolutionary dynamics

**Fig 3:**
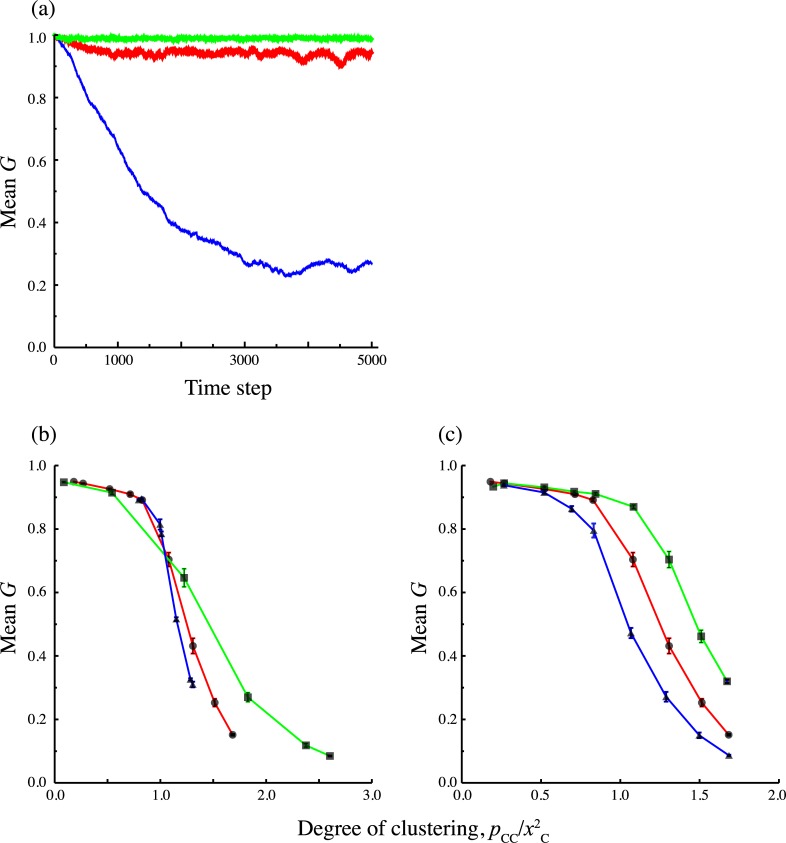
Results of Monte-Carlo simulation. Parameters that are not denoted below are the same as in Fig 2A, *x*_*C*_ = 0.5, *α* = 1, *β*_*G*_ = *β*_*L*_ = 10. (a) Examples of evolutionary trajectories of mean proportion of global infection *G*. The red line denotes results for *p*_*CC*_ = 0.25, the green line for *p*_*CC*_ = 0.1, and the blue line for *p*_*CC*_ = 0.4. (b)-(c) Dependence of evolutionally outcomes after 5,000 time step simulation. We conduct 20 simulations for each value of $p_{CC}/x_{C}^{2}$ (horizontal axis), and the long-term average of the population mean *G* are shown (vertical axis). Bars denotes the standard deviation. (b) The red line denotes results for *x*_*C*_ = 0. 5, the green line for *x*_*C*_ = 0. 4, and the blue line for *x*_*C*_ = 0. 7. (c) The red line denotes the results for *β*_*G*_ = *β*_*L*_ = 10, the green line for *β*_*G*_ = 10, *β*_*L*_ = 8, and the blue line for *β*_*G*_ = 8, *β*_*L*_ = 10.

The mean *G* in the population quickly converges to a certain level, and fluctuates around that level (Fig 3A). As long as we use the same parameters, the evolutionary outcomes are similar to each other regardless of the initial condition and the number of trials. We found that evolutionary branching never occurs and the distribution of strains is always unimodal (see S2 Fig). Fig 3B and 3C shows evolutionary outcomes with changing the degree of spatial correlation $p_{CC}/x_{C}^{2}$ like Fig 2C and 2D, where 20 trials are conducted for each parameter set. In general, the results are similar to the pair approximation, notably, 1) high $p_{CC}/x_{C}^{2}$ prefers local infection, 2) evolutionary outcome depends on both $p_{CC}/x_{C}^{2}$ and *x*_*C*_, and 3) increasing *x*_*C*_ promotes the local infection.

The difference between the results from pair approximation (Fig 2C and 2D) and those from simulations (Fig 3B and 3C) is that the mean value of *G* in the simulations does not converge to extreme values (the completely global or the completely local). This may be because the population is always polymorphic as a result of mutations. In addition, there are two other reasons for the region in which the completely local is the ESS according to the pair approximation. The first reason is the effect of finite population size. Especially, since the completely local strain spreads only in a contiguous cluster, the number of available hosts is smaller than for other strains. Therefore, diffusing to other clusters becomes adaptive. The second one is the limitation of pair approximation. In this approximation, we approximate *q*_*σ*/*σ*′*σ*″_ the conditional probability that a randomly chosen nearest of a *σ*'*σ*'' pair has a *σ* site by *q*_*σ*/*σ*′_ the conditional probability that a randomly chosen nearest neighbor of *σ*' site is a *σ* site. When viruses are too biased toward local infection, infected cells tend to form a large cluster and the approximation does not work well. For these reasons, there is a discrepancy between pair approximation and simulation.

**Fig 4:**
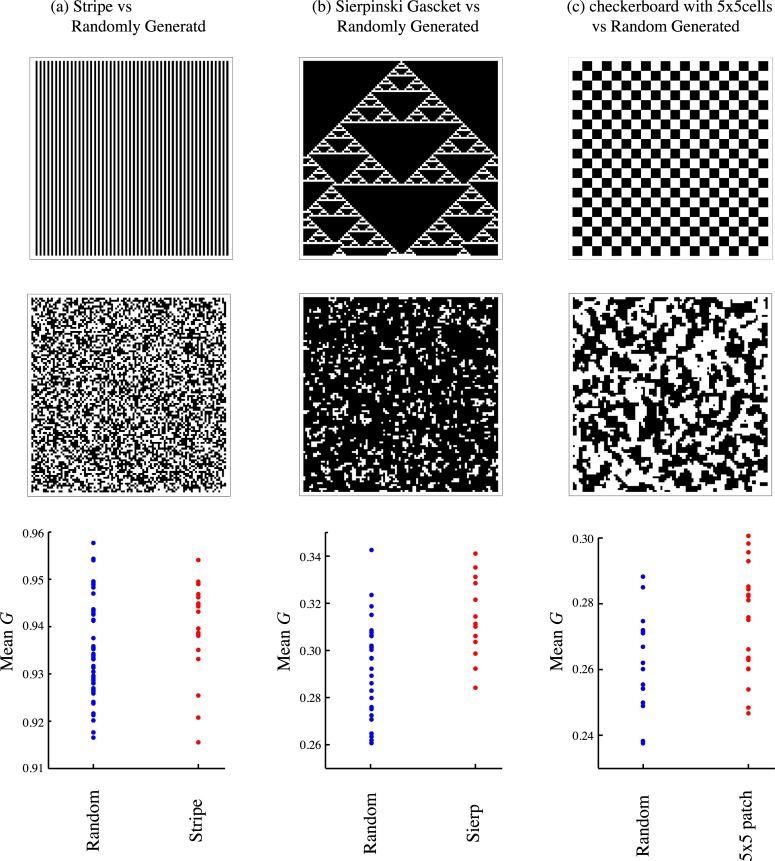
Evolutionary outcomes on randomly and deterministically generated structures (Top) The deterministically generated spatial structures, where target cells are shown by white. (a) The vertical stripe pattern with the line width 1, (b) the Sierpinski gasket, (c) checkerboard pattern with each cell sized as 5x5. (Middle) The corresponding randomly generated spatial patterns that have the same global and pair densities as those in the top row. (a) *x*_*C*_ = 0.5 and *p*_*CC*_ = 0.25, (b) *x*_*C*_ = 0.16 and *p*_*CC*_ = 0.07, (c) *x*_*C*_ = 0.5 and *p*_*CC*_ = 0.4. (Bottom) Comparison of the results of Monte Carlo simulations for the evolution of the fraction of global infection (*G*). For each spatial structure, 20 simulation runs are conducted, and the resulting population mean fractions of global infection are plotted as dots. In randomly generated structures, the spatial configurations are regenerated in each simulation run. Other parameters are *α* = 1, *β*_*G*_ = *β*_*L*_ = 10.

To check whether these results are specific to our method of generating a spatial structure, we also conducted the same evolutionary simulations on the several different deterministically generated structures each having the same global and pair densities (*x*_*C*_ and *p*_*CC*_) as those in randomly generated structure. Fig 4 shows the comparison of results between randomly and deterministically generated structures. These results suggest the robustness of our results on the evolution of local and global infections based on the randomly generated spatial configurations for given singlet and doublet densities, *x*_*C*_ and *p*_*CC*_.

### Nonlinear trade-off

**Fig 5:**
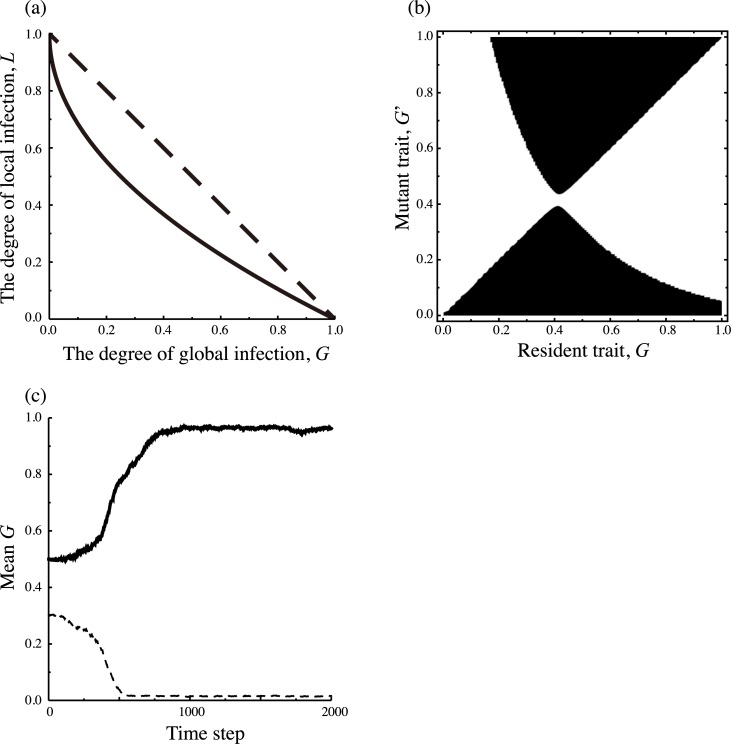
Evolution of the fraction of global infection with a non-linear trade-off. (a) A linear trade-off between the degree of global infection *G* (horizontal axis) and that of local infection *L* (vertical axis) assumed so far (dashed curve), and a non-linear trade-off examined here (solid curve), where the local infection decreases in proportional to $G^{0.5}:\;L=1-\sqrt{G}$. (b) Pairwise invasibility plot with *x*_*C*_ = 0.5, *p*_*CC*_ = 0.4, *α* = 1, *β*_*G*_ = *β*_*L*_ = 10. The black region shows that a mutant strain can invade the resident population, indicating that the evolutionary singular point around *G* = 0.4 is an evolutionary repeller. (c) Examples of evolutionary trajectory for the mean degree of global infection observed in Monte Carlo simulations starting from two different initial mean values of *G*(*G* = 0.3 and *G* = 0.5).

In the above sections, we assumed a linear trade-off, that is, the proportion of local infection decreases at the same amount as the proportion of global infection *G* is increased. However, this should not be always the case in reality. When the local infection decreases in proportion to *G*^0.5^, the result is quite different (Fig 5). In this case, the PIP shows bistability in which the evolutionary outcome depends on an initial state (Fig 5B). This prediction by pair approximation is also confirmed by simulation (Fig 5C).

## Discussion

In this paper, we investigated the effect of population structure on an evolutionarily stable proportion of global infection. Before considering the effect, we have obtained the endemic condition of a virus strain in the SIS model on a lattice space using pair approximation. For the extreme cases (completely global or completely local strain), the endemic condition is consistent with previous studies [18,21]. For the intermediate global infection rate, the condition shows that the strain using both global and local infection can survive with the parameter sets with which the extreme strains cannot. For example, when the global density of target cells is too low to persist for completely global infection, high degree of clustering of target cells promotes the survival of a strain using local infection.

In fact, the invasibility analysis by pair approximation and the Monte-Carlo simulation show that evolutionarily stable strategies or evolutionary outcomes are strongly dependent on the global density of target cells and their degree of clustering. As target cells become aggregated, higher proportion of local infection is selected. This tendency can be seen in the negative slopes in the relationship between ESS *G* and the spatial aggregation measure $p_{CC}/x_{C}^{2}$ in Fig 2. This effect seems stronger at high global density of target cells because the slope of high *x*_*C*_ is steeper than that of low *x*_*C*_ (Fig 2C). When the local density of target cells is high, spread through local infection is easier for finding the next susceptible cell compared to global infection. Thus, a virus that uses local infection may predominantly be efficient in spreading and that trait is selected. However, there is also a difference between invasibilty analysis and simulation, whereby the former predicts the completely local strain can be ESS in some range but the latter does not. This discrepancy may be attributed to the limitation of pair approximation. In the simulation of evolutionary dynamics, we can generate different spatial structures that have the same first and second moment (global and pair densities) but have different higher order moments. In spite of these variations in spatial structure, the evolutionary outcomes were very similar (Figs 2, 3 and 4). Therefore, we conclude that global and pair densities are enough to explain outcome of evolution of global or local infection, and that parameters which describe higher order configuration do not affect results.

We also checked the dependence of ESS on the parameters that define infection dynamics. When the ratio *β*_*G*_/*β*_*L*_ is changed with fixing $p_{CC}/x_{C}^{2}$, the ESS is adjusted to use more efficient pathway (for example, an ESS *G* for *β*_*G*_/*β*_*L*_ > 1 is larger than that for *β*_*G*_ = *β*_*L*_; Fig 2D). The analytical result show that the magnitude of *β*_*G*_/*β*_*L*_ relative to $p_{CC}^{2}/x_{C}$ also affects whether or not the completely global strain can be an ESS. When the degree of clustering $p_{CC}/x_{C}^{2}$ is smaller than *β*_*G*_/*β*_*L*_, the completely global infection becomes ESS. If *β*_*G*_ = *β*_*L*_, the boundary is at complete spatial randomness (CSR). If *β*_*G*_ < *β*_*L*_, using some local infection can be evolutionarily stable even if targets cells are more uniformly distributed than CSR. The effect of increasing recover rate *α* is against to favor local infection. This result is apparently counter-intuitive because high recovery rate would mitigate the self-shading effect [14] and hence local infection would be more efficient. However, high recovery rate increases the density of disease-free clusters, which makes global infection far beneficial in accessing the isolated clusters.

The evolution of global vs local spread is also addressed in some studies in ecology and epidemiology [14–17]. In the cited studies, spatial structure is either not considered at all or limited only to some patchy distribution. Self-organized spatial structures are not definitely controlled or parameterized during simulation. Thus, our model defines spatial structure at first, and examines the dependences of evolutionary outcomes on spatial parameters. The other difference is the effect of "self-shading". If infected individuals are clustered, the number of susceptible individuals available for local infection decreases. Kamo and Boots [14] assumed that infected individuals will die and vacant sites will be covered only by host local reproduction, which has a strong effect against local infection. In contrast, our model assumes SIS model in which an infected cell changes its state to susceptible and the spatial structure is mainly caused by definition. Therefore, self-shading effect is weaker than the previous studies and our analytical results show the suitable parameter region where the completely local strain can be an ESS. In terms of dynamics on artificially organized spatial structure, Hiebeler [18,19] modeled the competition between species using different proportion of global and local reproduction. In such models, the pairwise invasibility between resident and invader (or mutant) was checked, but the evolution of the trait after invasion was not considered. Here we used similar model for the within-host viral evolution and applied adaptive dynamics framework. It suggests that invasion has the possibility of replacement by mutants and this replacement drives the evolution of traits in a population. Applying adaptive dynamics is justified by the assumption that we focus on viruses that cause persistent infection. Such persistent viruses may have sufficient time for the within-host evolution and the adaptive dynamics can be applied. There should be conflicts between the aims for the short-term increase within a host and that for the long-term spreads between hosts. However, the combined effects of these conflicting selection processes is beyond the scope of the present paper.

As in the previous models [14–17], we assumed a trade-off between global and local infection. The cost of local infection in our model is superinfection caused by the retention of virions on the infected cell surface, which is also assumed in [15,16]. The importance of superinfection is inferred by the fact that various viruses have a mechanism to avoid superinfection promoted by local infection [24–29].

### Ecological implication

From an ecological point of view, our model can be applied for considering the evolution of long and short dispersal. Target cells correspond to habitats for animals or plants, with S sites and I sites corresponding to unoccupied and occupied sites, and non-target cells representing unsuitable sites to settle. According to the endemic condition (6), the persistence of a species with some local colonization rate depends on the spatial structure. Our results, if applied to a conservation biological setting, suggest that, even if we conserve the abundance of habitats for an endangered species, extinction might occur only due to the change in spatial arrangement of habitats. In terms of the evolution of dispersal distance, previous studies add other settings such as a trade-off between survivability and dispersal range [30], the population dynamics in local patches [31], kin selection [32], the existence of pests [33], or the disturbance structure [34,35]. However, the effect of spatial structure per se has not been clarified yet. We therefore checked how spatial structure affects the evolution of shot and long dispersal. Our results suggest that the ESS proportion of short dispersal depends not only on the degree of clustering but also on the density of habitats per se. Short dispersal is selected when habitats are clustered, and this tendency is strong when the abundance of habitats is high.

Our model is also regarded as a model of dispersal rate evolution that considers how much an offspring should disperse beyond its natal patch if we arrange habitats like patches. In this interpretation, local infection denotes staying in a natal patch and global infection represents outgoing from a natal patch. The infection rates *β*_*G*_ and *β*_*L*_ correspond to survival rate of outgoing and staying individuals, respectively. The conventional result in this situation suggests that even if the survival rate of an outgoing individual (*β*_*G*_) is much lower than that of a staying individual (*β*_*L*_), at least half of all offsprings should go out [36]. Pair approximation in the present study does not predict this result but this is clearly due to the limit of pair approximation. Actually, Monte-Carlo simulation shows that the completely local strategy is never selected. In addition to the previous result, we suggest that even if the survival rate of outgoing individual is higher than that of staying one (*β*_*G*_ > *β*_*L*_), there is a parameter region in which some offspring should be left due to high $p_{CC}/x_{C}^{2}$. Although a similar result has been suggested [37], the reasoning differs to our study. The previous result is due to the variation of patch quality, which means that an individual born in a good patch should leave its offspring in a natal patch. In our model, high clustering of habitats prefers leaving offsprings in a natal patch without assuming differences in patch quality. When the habitats are highly clustered ($p_{CC}/x_{C}^{2}>1$), the probability of finding new habitat is higher for local dispersal (*q*_*C/C*_ = *p*_*CC*_/*x*_*C*_) than for global dispersal (*x*_*C*_). Therefore, the advantage of finding a new habitat can outweigh the disadvantage of low survival rate.

Our model predicts the evolutionarily stable dispersal strategy only but some previous models suggest evolutional branching of dispersal rate [38,39] or evolutionary bistability in which the evolutionary outcome differs in initial state [40]. In general, branching or bistability may occur when the fitness of a phenotype depends on the frequencies of other existing phenotypes and possible phenotypes have a proper trade-off [41,42]. In our model, there is a trade-off between global and local infection. The reason why we only observe ESS is that the linear trade-off is not suitable for causing evolutionary branching or bistability. In fact, if we assume nonlinear trade-off between global and local infection is assumed, we can observe the evolutionary bistability (Fig 5).

### Virological implication

Our model suggests that spatial structure has an important role, while it is not commonly considered in the field of virology. In *in vitro* experimental cases, two-dimensional cell culture is commonly used and this condition is similar to our model. Hence, there is a possibility that the evolution of global and local infection can occur. In an example of culture of measles viruses (MVs), a higher level of local infection was selected for in continuing passages [43], indicating the emergence of mutant viruses with a high ability to induce membrane fusion *in vitro*. When the evolution of global or local infection occur, other viral traits like virulence will evolve as shown in Boots and Sasaki [8]. They analytically showed that a lower virulence is predicted as infection becomes more local. The importance of local infection in the evolution of influenza viruses is shown experimentally [3,4]: cell-to-cell transmission promotes a faster expansion of the diversity of virus quasispecies and may facilitate viral evolution and adaptation when influenza viruses’ neuraminidases are inhibited and virus release from infected cells is suppressed [3,4]. Therefore, we emphasize the relationship between the spatial distribution of target cells and the evolution of viral infection mode when studying *in vitro* infectious dynamics. If we manipulate cell density and the efficiency of viral transmission by antibodies, viruses that have favorable level of global and local infection may be obtained.

In a host body, it is rare that cells distribute like two-dimensional cell culture systems except for epithelia. Epithelial cells form a continuous sheet and they may be different in susceptibility because of surface molecule expression, response to interferons and other immune cell activities. Therefore, epithelia can be a place where evolution of cell-to-cell infection can occur, and in fact, some viruses are known to have an ability for cell-to-cell infection in epithelia like MVs [44] (for other examples, see Table 1 in [5]). In contrast, influenza viruses can also infect epithelial cells but the evolution of local infection is not known. The reason may be the short length of infection period; influenza infection period can be as short as a week but some viruses like HIV survive in the host body for a long time. Such persistent viruses may have sufficient time for the within-host evolution and the adaptive dynamics framework can be applied. Since MVs also have an ability to establish persistent infection, these viruses may also have a chance to evolve efficient cell-to-cell infection in the host body.

It has been shown that cell-to-cell viral transmission through virological synapse occurs in retroviruses such as human T-lymphotropic virus type 1 (HTLV-1) [45] and HIV [46]. This process is thought to have important role because the contribution of cell-to-cell infection on HIV spread is estimated to be equal to or more that of cell-free infection by comparing static and shaking culture conditions [47,48]. Since infected cells can move in the lymphoid tissue and find a connection to susceptible cells, the spatial viscosity of the infected target cells in the lymph nodes should be weakened in these viruses. However, there would remain some non-random correlation of uninfected target cells ($p_{CC}/x_{C}^{2}>1$) because of the locality of T cells in the lymph nodes, and this could favor cell-to-cell transmission over cell-free transmission in retroviruses too. Therefore, we suggest new conditions that evolutionarily promote cell-to-cell infection in those viruses: highly localized distribution of target cells in the lymphoid tissues ($p_{CC}/x_{C}^{2}>1$). These points have not been suggested in the previous theoretical study [16].

Our model may explain the emergence of mutant MVs that are isolated from patients of subacute sclerosing panencephalitis (SSPE). These viruses have mutations that provide high ability of cell fusion (i.e. high level of local infection) [49] and can infect central nervous system cells, while wild type MVs cannot [43,49]. Since MVs can spread in a cell-to-cell manner between epithelial cells, epithelia is a candidate place in which the evolution of local infection occurs. Another possibility is lymph nodes because the main target cell of MVs is SLAM (signaling lymphocytic activation molecule, also known as CD150) expressing immune cells such as T and B cells etc. [50,51]. As discussed in the case of HIV, concentrating target cells in the lymph nodes satisfies the condition under which local infection is selected. Consequently, MVs are prone to evolve local infection in a host body and to gain the ability to infect cells of central nervous system but how MVs reach the central nervous system remains unknown.

In conclusion, our results suggest that the mode of viral spread, global or local infection, may undergo adaptive evolutionary change *in vitro* and *in vivo*. In the future, we can consider more realistic situation such as evolutionary dynamics in three-dimensional space or the repulsion of superinfecting virions that attenuates self-shading effect [29]. In order to examine the emergence of mutant virus or the evolution of virulence, we need to take into account the fact that the mode of infection itself is subject to selection.

## Supporting information

S1 Text Stability analysis of equilibrium. Appendix A shows the case with a single strain and Appendix B shows the case with resident and mutant strains.
(DOCX)

S1 Fig Dependence of ESS *G* on recovery rate *α* with *β*_*G*_ < *β*_*L*_. For all lines, fixed parameters are *x*_*C*_ = 0.5, *β*_*G*_ = 8, *β*_*L*_ = 10. The red line denotes the evolutionarily stable (ESS) proportion of global infection *G* for *p*_*CC*_ = 0.2, the black line denotes that for *p*_*CC*_ = 0.25 the green line denotes that for *p*_*CC*_ = 0.3, and the blue line denotes that for *p*_*CC*_ = 0.33.
(EPS)

S2 Fig Distributions of strains in Monte-Carlo simulation. The horizontal axis is the proportion of global infection of strains and the vertical axis denotes their global densities. Blue represents *p*_*CC*_ = 0.25, red represents *p*_*CC*_ = 0.4. Other parameters are *x*_*C*_ = 0.5, *α* = 1, *β*_*G*_ = *β*_*L*_ = 10.
(EPS)
