# Supplementary material for: The role of spatial heterogeneity in the evolution of local and global infections of viruses
Source: PLoS Comput Biol. 2018 Jan 25;14(1):e1005952. doi: 10.1371/journal.pcbi.1005952 (PMC5800656; doi:10.1371/journal.pcbi.1005952)
Supplement: S1 Text — Appendix A shows the case with a single strain and Appendix B shows the case with resident and mutant strains. (DOCX) [file pcbi.1005952.s001.docx]

**Appendix A: Dynamics of a single strain**

Let consider the dynamics of pair densities for two neighboring target cells. According to equations (3), $\phi$ includes global infection rate and local infection rate from an infected cell in one of the other 3 neighboring sites. $\psi$ shows local infection rate between cells that we are focusing on. Then, the changes of state by infection are: from *SS* to *SI* at rate $\phi$, and from *SI* to *II* at rate $\phi+\psi$. The changes by recovery are opposite direction at rate $\alpha$. Thus, the dynamics are

|  | $\frac{dp_{SS}}{dt}=2\left( -\phi p_{SS}+\alpha p_{SI} \right),$ | (A1a) |
| --- | --- | --- |
|  | $\frac{dp_{SI}}{dt}={-\left( \phi+\psi+\alpha\right)p}_{SI}+\phi p_{SS}+\alpha p_{II},$ | (A1b) |
|  | $\frac{dp_{II}}{dt}=2\left\{ \left( \phi+\psi\right)p_{SI}-\alpha p_{II} \right\}.$ | (A1c) |

In the same manner, the dynamics of pair densities for target and non-target cells are

|  | $\frac{dp_{SO}}{dt}=-\phi p_{SO}+\alpha p_{IO},$ | (A1d) |
| --- | --- | --- |
|  | $\frac{dp_{IO}}{dt}=\phi p_{SO}-\alpha p_{IO}.$ | (A1e) |

Since $x_{I}=p_{SI}+p_{II}+p_{IO}$, the dynamics of $x_{I}$ is obtained by summing up Equation (A1b), (A1c) and (A1e),

|  | $\frac{dx_{I}}{dt}=\left\{ gx_{S}+\left( l+\psi\right)q_{S/I}-\alpha\right\}x_{I}.$ | (A1f) |
| --- | --- | --- |

If we choose $x_{I},$ $p_{SI}$ and $p_{IO}$ as three independent variables, other variables should be defined, that is, $x_{S}=x_{C}-x_{I}$, $q_{S/I}=p_{SI}/x_{I}$, $p_{SO}=x_{C}-p_{CC}-p_{IO}$, $p_{SS}=x_{C}-x_{I}-p_{SI}-\left( x_{C}-p_{CC}-p_{IO} \right)=p_{CC}-x_{I}-p_{SI}+p_{IO}$, $p_{II}=x_{I}-p_{SI}-p_{IO}$. Then, the dynamics of three variables are

|  | $\frac{dx_{I}}{dt}=\left\{ g\left( x_{C}-x_{I} \right)+\left( l+\psi\right)\frac{p_{SI}}{x_{I}}-\alpha\right\}x_{I},$ | (A2a) |
| --- | --- | --- |
|  | $\frac{dp_{SI}}{dt}=-\left\{ 2\left( gx_{I}+l\frac{p_{SI}}{x_{C}-x_{I}} \right)+\psi+2\alpha\right\}p_{SI}+\alpha\left( x_{I}-p_{IO} \right)+\left( gx_{I}+l\frac{p_{SI}}{x_{C}-x_{I}} \right)\left( p_{CC}-x_{I}+p_{IO} \right),$ | (A2b) |
|  | $\frac{dp_{IO}}{dt}=\left( gx_{I}+l\frac{p_{SI}}{x_{C}-x_{I}} \right)\left( x_{C}-p_{CC}-p_{IO} \right)-\alpha p_{IO}.$ | (A2c) |

*Calculation of basic reproductive number*

Linearized dynamics around the disease free equilibrium of which $x_{I}=p_{SI}=p_{IO}=0$ is obtained from equations (A2),

|  | $\frac{d}{dt}\left( \begin{matrix} x_{I} \\ p_{SI} \\ p_{IO} \end{matrix} \right)=\left( \begin{matrix} gx_{C}-\alpha& l+\psi& 0 \\ gp_{CC}+\alpha& \frac{p_{CC}}{x_{C}}l-\psi-2\alpha& -\alpha\\ g\left( x_{C}-p_{CC} \right) & \left( 1-\frac{p_{CC}}{x_{C}} \right)l & -\alpha\end{matrix} \right)\left( \begin{matrix} x_{I} \\ p_{SI} \\ p_{IO} \end{matrix} \right).$ | (A3) |
| --- | --- | --- |

The local stability of the trivial solution of this system is lost if the determinant of the Jacobian matrix in the right hand side of equations (A3) becomes positive. Then, the condition is obtained as shown in inequality (6).

According to Diekmann et al. [1], the next generation matrix is calculated from (A3). The Jacobian matrix ***J*** is dissolved by $\boldsymbol{J}=\boldsymbol{F}-\boldsymbol{V}$, where ***F*** and ***V*** describes the infection and recovery (transition) processes respectively, and

|  | $\boldsymbol{F}=\left( \begin{matrix} gx_{C} & l+\psi& 0 \\ gp_{CC} & \frac{p_{CC}}{x_{C}}l-\psi& 0 \\ g\left( x_{C}-p_{CC} \right) & \left( 1-\frac{p_{CC}}{x_{C}} \right)l & 0 \end{matrix} \right),$ | (A4a) |
| --- | --- | --- |
|  | $\boldsymbol{V}=\left( \begin{matrix} \alpha& 0 & 0 \\ -\alpha& 2\alpha& \alpha\\ 0 & 0 & \alpha\end{matrix} \right).$ | (A4b) |

The next generation matrix is given by $\boldsymbol{F}\cdot\boldsymbol{V}^{-1}$, that is,

|  | $\boldsymbol{F}\cdot\boldsymbol{V}^{-1}=\left( \begin{matrix} \frac{gx_{C}}{\alpha}+\frac{l+\psi}{2\alpha} & \frac{l+\psi}{2\alpha} & -\frac{l+\psi}{2\alpha} \\ \frac{gp_{CC}}{\alpha}+\frac{\frac{lp_{CC}}{x_{C}}-\psi}{2\alpha} & \frac{\frac{lp_{CC}}{x_{C}}-\psi}{2\alpha} & -\frac{\frac{lp_{CC}}{x_{C}}-\psi}{2\alpha} \\ \frac{g\left( x_{C}-p_{CC} \right)}{\alpha}+\frac{l\left( 1-\frac{p_{CC}}{x_{C}} \right)}{2\alpha} & \frac{l\left( 1-\frac{p_{CC}}{x_{C}} \right)}{2\alpha} & -\frac{l\left( 1-\frac{p_{CC}}{x_{C}} \right)}{2\alpha} \end{matrix} \right).$ |  |
| --- | --- | --- |

Finally, we have the basic reproductive number *R*_0_ as the maximum eigenvalue of the next generation matrix (the result is equation (6) in the main text).

*Calculation of Equilibrium*

Let the densities at endemic equilibrium of virus be $\hat{p}_{SS}$, $\hat{p}_{SI}$, $\hat{p}_{IO}$, $\hat{p}_{II}$. From equations (A1), the following relationships should be satisfied,

|  | $\frac{\hat{\phi}}{\alpha}=\frac{\hat{p}_{SI}}{\hat{p}_{SS}}=\frac{\hat{p}_{IO}}{\hat{p}_{SO}}\equiv\xi,$ | (A5a) |
| --- | --- | --- |
|  | $\frac{\hat{\phi}+\psi}{\alpha}=\frac{\hat{p}_{II}}{\hat{p}_{SI}}\equiv\xi+\eta,$ | (A5b) |

where symbols with hat denote equilibrium density or equilibrium value of $\phi$. From the first equality of (A4a), (A4b), and $p_{SS}+2p_{SI}+p_{II}=p_{CC}$,

|  | $\hat{p}_{SS}=\frac{p_{CC}}{F},$ | (A6a) |
| --- | --- | --- |
|  | $\hat{p}_{SI}=\frac{{\xi p}_{CC}}{F},$ | (A6b) |
|  | $\hat{p}_{II}=\frac{\left( \xi^{2}+\xi\eta\right)p_{CC}}{F},$ | (A6c) |

where $F=\left( 1+\xi\right)^{2}+\xi\eta$. From the second equality of (A5a) and $p_{SO}+p_{IO}=x_{C}-p_{CC}$,

|  | $\hat{p}_{SO}=\frac{x_{C}-p_{CC}}{1+\xi},$ | (A6d) |
| --- | --- | --- |
|  | $\hat{p}_{IO}=\frac{\xi\left( x_{C}-p_{CC} \right)}{1+\xi}.$ | (A6e) |

Thus, if we calculate $\xi$, equilibrium densities are obtained. From equations (A6), we have global densities at equilibrium,

|  | $\hat{x}_{S}=\hat{p}_{SS}+\hat{p}_{SI}+\hat{p}_{SO}=\frac{\left( 1+\xi\right)p_{CC}}{F}+\frac{x_{C}-p_{CC}}{1+\xi}$ $=\frac{Fx_{C}-\xi\eta p_{CC}}{\left( 1+\xi\right)F},$ | (A7a) |
| --- | --- | --- |
|  | $\hat{x}_{I}=\hat{p}_{II}+\hat{p}_{SI}+\hat{p}_{IO}=\frac{\left( \xi+\xi^{2}+\xi\eta\right)p_{CC}}{F}+\frac{\xi\left( x_{C}-p_{CC} \right)}{1+\xi}$ $=\frac{\xi Fx_{C}+\xi\eta p_{CC}}{\left( 1+\xi\right)F}.$ | (A7b) |

$\xi$ is calculated from its definition,

|  | $\xi=\frac{\phi}{\alpha}=\frac{g}{\alpha}\hat{x}_{I}+\frac{l}{\alpha}\frac{\hat{p}_{SI}}{\hat{x}_{S}}$ $=\frac{g}{\alpha}\frac{\xi Fx_{C}+\xi\eta p_{CC}}{\left( 1+\xi\right)F}+\frac{l}{\alpha}\frac{\xi p_{CC}}{F}\frac{\left( 1+\xi\right)F}{Fx_{C}-\xi\eta p_{CC}}$ |  |
| --- | --- | --- |

or

|  | $1=\frac{g}{\alpha}\frac{Fx_{C}+\eta p_{CC}}{\left( 1+\xi\right)F}+\frac{l}{\alpha}\frac{\left( 1+\xi\right)p_{CC}}{Fx_{C}-\xi\eta p_{CC}}$ $=\frac{\beta_{G}}{\alpha}G\frac{Fx_{C}+\eta p_{CC}}{\left( 1+\xi\right)F}+\frac{\beta_{L}\left( 1-\theta\right)}{\alpha}\left( 1-G \right)\frac{\left( 1+\xi\right)p_{CC}}{Fx_{C}-\xi\eta p_{CC}}.$ | (A8) |
| --- | --- | --- |

For a given value of *G*, $\xi$ is only numerically calculated from equation (A8). When *G* = 1, calculating an equilibrium is much easier. Equation (A8) becomes,

|  | $\frac{{\beta_{G}x}_{C}}{\alpha\left( 1+\xi\right)}=1,$ |  |
| --- | --- | --- |

Then, we can obtain the equilibrium of completely global virus,

|  | $\xi=\frac{\beta_{G}x_{C}}{\alpha}-1,$ | (A9a) |
| --- | --- | --- |
|  | $\hat{x}_{I}=x_{C}-\frac{\alpha}{\beta_{G}},$ | (A9b) |
|  | $\hat{p}_{SI}=\left( 1-\frac{\alpha}{\beta_{G}x_{C}} \right)\frac{\alpha}{\beta_{G}x_{C}}p_{CC},$ | (A9c) |
|  | $\hat{p}_{IO}=\left( 1-\frac{\alpha}{\beta_{G}x_{C}} \right)\left( x_{C}-p_{CC} \right).$ | (A9d) |

**Appendix B: Dynamics of resident and mutant**

With a mutant strain is denoted by subscript *J*, let its proportion of global infection be *G'*. Like equations (3), the probability that the susceptible cell in a *SJ* pair gets infected per unit time interval is $\phi'+\psi'$, that is,

|  | $\phi'=\beta_{G}G'x_{J}+\beta_{L}\left( 1-G^{'} \right)\left( 1-\theta\right)q_{J/S}=g'x_{J}+l'\frac{p_{SJ}}{x_{S}},$ | (B1a) |
| --- | --- | --- |
|  | $\psi'=\beta_{L}\left( 1-G' \right)\theta.$ | (B1b) |

There are six states, *SS*, *SI*, *SJ*, *II*, *IJ* and *JJ* in a pair of target cells, and the sum of their densities is equal to a constant $p_{CC}$:

|  | $p_{SS}+p_{SI}+p_{SJ}+p_{II}+p_{IJ}+p_{JJ}=p_{CC}.$ |  |
| --- | --- | --- |

The dynamics of these states are

|  | $\frac{dp_{SS}}{dt}=2\left\{ -\left( \phi+\phi^{'} \right)p_{SS}+\alpha\left( p_{SI}+p_{SJ} \right) \right\},$ | (B2a) |
| --- | --- | --- |
|  | $\frac{dp_{SI}}{dt}={-\left( \phi+\phi'+\psi+\alpha\right)p}_{SI}+\phi p_{SS}+\alpha\left( p_{II}+p_{IJ} \right),$ | (B2b) |
|  | $\frac{dp_{SJ}}{dt}={-\left( \phi+\phi'+\psi'+\alpha\right)p}_{SJ}+\phi^{'}p_{SS}+\alpha\left( p_{JJ}+p_{IJ} \right),$ | (B2c) |
|  | $\frac{dp_{II}}{dt}=2\left\{ \left( \phi+\psi\right)p_{SI}-\alpha p_{II} \right\},$ | (B2d) |
|  | $\frac{dp_{IJ}}{dt}={\phi p}_{SJ}+\phi^{'}p_{SI}-2\alpha p_{IJ},$ | (B2e) |
|  | $\frac{dp_{JJ}}{dt}=2\left\{ \left( \phi'+\psi' \right)p_{SJ}-\alpha p_{JJ} \right\}.$ | (B2f) |

On the other hand, there are three states, *SO*, *IO*, and *JO* in a pair of target and non-target cells, and the sum of their densities equal to a constant $x_{C}-p_{CC}$:

|  | $p_{SO}+p_{IO}+p_{JO}=x_{C}-p_{CC}.$ |  |
| --- | --- | --- |

The dynamics of these states are

|  | $\frac{dp_{SO}}{dt}=-\left( \phi+\phi^{'} \right)p_{SO}+\alpha\left( p_{IO}+p_{JO} \right),$ | (B2g) |
| --- | --- | --- |
|  | $\frac{dp_{IO}}{dt}={\phi p}_{SO}-\alpha p_{IO},$ | (B2h) |
|  | $\frac{dp_{JO}}{dt}={\phi'p}_{SO}-\alpha p_{JO}.$ | (B2i) |

We choose $x_{I}$, $p_{SI}$, $p_{IO}$, $x_{J}$, $p_{SJ}$, $p_{IJ}$, and $p_{JJ}$ as seven independent variables whose dynamics are shown in equations (5) in the main text. Now we consider the invasibility of a mutant strain in the endemic equilibrium of a resident strain. Since the densities of residents are not changed at equilibrium, we only have to consider the dynamics of densities with mutants $x_{J}$, $p_{SJ}$, $p_{IJ}$, and $p_{JJ}$. The linearized dynamics around resident equilibrium $\left( x_{I},p_{SI},p_{IO},x_{J},p_{SJ},p_{IJ},p_{JJ} \right)=\left( \hat{x}_{I},\hat{p}_{SI},\hat{p}_{IO},0,0,0,0 \right)$ are

|  | $\frac{d}{dt}\left( \begin{matrix} x_{J} \\ p_{SJ} \\ p_{IJ} \\ p_{JJ} \end{matrix} \right)=\left( \begin{matrix} g^{'}\hat{x}_{S}-\alpha& l^{'}+\psi' & 0 & 0 \\ g^{'}\hat{p}_{SS} & X-\psi^{'}-\alpha& \alpha& \alpha\\ g^{'}\hat{p}_{SI} & Y & -2\alpha& 0 \\ 0 & 2\psi' & 0 & -2\alpha\end{matrix} \right)\left( \begin{matrix} x_{J} \\ p_{SJ} \\ p_{IJ} \\ p_{JJ} \end{matrix} \right)$ | (B4) |
| --- | --- | --- |

where $\hat{x}_{S}=x_{C}-\hat{x}_{I}$, $\hat{p}_{SS}=\hat{x}_{S}-\hat{p}_{SI}-\hat{p}_{SO}$, and

|  | $X=l^{'}\frac{\hat{p}_{SS}}{\hat{x}_{S}}-\hat{\phi}=\frac{l^{'}\hat{p}_{SS}}{\hat{x}_{S}}-g\hat{x}_{I}-l\frac{\hat{p}_{SI}}{\hat{x}_{S}},$ |  |
| --- | --- | --- |
|  | $Y=l^{'}\frac{\hat{p}_{SI}}{\hat{x}_{S}}+\hat{\phi}=l^{'}\frac{\hat{p}_{SI}}{\hat{x}_{S}}+g\hat{x}_{I}+l\frac{\hat{p}_{SI}}{\hat{x}_{S}}.$ |  |

The invasibility of mutants can be checked by the sign of the dominant eigenvalue of Jacobian matrix in equation (B4). Let Jacobian matrix be ***A***, then, its characteristic equation is

|  | $\left\vert\begin{matrix} g^{'}\hat{x}_{S}-\alpha-\lambda& l^{'}+\psi' & 0 & 0 \\ g^{'}\hat{p}_{SS} & X-\psi^{'}-\alpha-\lambda& \alpha& \alpha\\ g^{'}\hat{p}_{SI} & Y & -2\alpha-\lambda& 0 \\ 0 & 2\psi' & 0 & -2\alpha-\lambda\end{matrix} \right\vert=0,$ |  |
| --- | --- | --- |

where $\lambda$ is the eigenvalue. This equation is rewritten as

|  | $\left( \lambda+2\alpha\right)\left( \lambda^{3}+a_{1}\lambda^{2}+a_{2}\lambda+a_{3} \right)=0,$ | (B5) |
| --- | --- | --- |

where coefficients are

|  | $a_{1}=-\left( g^{'}\hat{x}_{S}+X-\psi^{'}-4\alpha\right),$ | (B6a) |
| --- | --- | --- |
|  | $a_{2}=-2\alpha\left( g^{'}\hat{x}_{S}+X-2\alpha\right)+\left( g^{'}\hat{x}_{S}-\alpha\right)\left( X-\psi^{'}-\alpha\right)-\alpha Y-g^{'}\hat{p}_{SS}\left( l^{'}+\psi' \right),$ | (B6b) |
|  | $a_{3}=\alpha\left( g'\hat{x}_{S}-\alpha\right)\left( 2X+Y-2\alpha\right)-\alpha g'\left( l^{'}+\psi^{'} \right)(\hat{p}_{SI}+2\hat{p}_{SS}).$ | (B6c) |

Whether mutants can invade or not is determined by the sign of the real part of the dominant eigenvalue and it should be zero when $G^{'}=G$. From equations (B6), when $G^{'}=G$, the real part of the dominant eigenvalue never becomes zero unless $a_{3}=0$. Therefore, we can conclude that the mutant can invade the resident population if and only if $a_{3}<0$ and $a_{3}=0$ when $G^{'}=G$. Substituting $X$ and $Y$ into equation (B6c),

|  | $a_{3}=\alpha\left( g'\hat{x}_{S}-\alpha\right)\left\{ \left( \hat{p}_{SI}+2\hat{p}_{SS} \right)\frac{l'}{\hat{x}_{S}}-\hat{\phi}-2\alpha\right\}-\alpha g'\left( l^{'}+\psi^{'} \right)(\hat{p}_{SI}+2\hat{p}_{SS}),$ |  |
| --- | --- | --- |
|  | $\frac{a_{3}}{\alpha}=-\left( \hat{p}_{SI}+2\hat{p}_{SS} \right)\left( g^{'}\psi^{'}+\alpha\frac{l^{'}}{\hat{x}_{S}} \right)-\left( g^{'}\hat{x}_{S}-\alpha\right)\left( \hat{\phi}+2\alpha\right).$ | (B7) |

When $G^{'}=G$, mutants become neutral to residents. Hence the dominant eigenvalue of Jacobian matrix become zero and we can obtain $a_{3}=0$, which leads

|  | $\hat{p}_{SI}+2\hat{p}_{SS}=-\frac{\hat{x}_{S}\left( g\hat{x}_{S}-\alpha\right)\left( \hat{\phi}+2\alpha\right)}{\alpha l+\psi g\hat{x}_{S}}.$ | (B8) |
| --- | --- | --- |

From equations (B7) and (B8),

|  | $\frac{a_{3}}{\alpha}=\left( \hat{\phi}+2\alpha\right)\left[ \frac{\left( g\hat{x}_{S}-\alpha\right)\left( \alpha l^{'}+\psi^{'}g^{'}\hat{x}_{S} \right)}{\alpha l+\psi g\hat{x}_{S}}-\left( g^{'}\hat{x}_{S}-\alpha\right) \right]$ $=\left( \hat{\phi}+2\alpha\right)\left( g\hat{x}_{S}-\alpha\right)\left[ \frac{\alpha l^{'}+\psi^{'}g^{'}\hat{x}_{S}}{\alpha l+\psi g\hat{x}_{S}}-\frac{g^{'}\hat{x}_{S}-\alpha}{g\hat{x}_{S}-\alpha} \right],$ |  |
| --- | --- | --- |

where the proportion of global infection of resident *G* should be $0\leq G<1$. According to equation (4a), $g\hat{x}_{S}<\alpha$ holds if $0\leq G<1$. Hence, the condition for $a_{3}<0$ is

|  | $\frac{\beta_{G}G^{'}\hat{x}_{S}-\alpha}{\beta_{G}G\hat{x}_{S}-\alpha}<\frac{1-G'}{1-G}\frac{\alpha\left( 1-\theta\right)+\theta\beta_{G}G^{'}\hat{x}_{S}}{\alpha\left( 1-\theta\right)+\theta\beta_{G}G\hat{x}_{S}},$ | (B9) |
| --- | --- | --- |

where $\hat{x}_{S}$ is obtained from equation (A7a). Of course, resident and mutant should satisfy the endemic condition (7). Practically, we numerically obtain $\hat{x}_{S}$ and check the condition (B9) to examine invasibility. Theoretically, the evolutionary singular *G* that is a candidate for ESS is obtained from

|  | $\left. \frac{\partial a_{3}}{\partial G^{'}} \right\vert_{G^{'}=G}=0.$ |  |
| --- | --- | --- |

Consider the case with the resident of completely global infection (*G* = 1). Equilibrium densities and $\xi$ are written in equations (A9), and for other densities

|  | $\hat{x}_{S}=\frac{\alpha}{\beta_{G}},$ |  |
| --- | --- | --- |
|  | $\hat{p}_{SS}=\left( \frac{\alpha}{\beta_{G}x_{C}} \right)^{2}p_{CC}.$ |  |

By substituting these quantities into equation (B7),

|  | $\frac{a_{3}}{\alpha}=-\left( \hat{p}_{SI}+2\hat{p}_{SS} \right)\left( g^{'}\psi^{'}+\alpha\frac{l^{'}}{\hat{x}_{S}} \right)-\left( g^{'}\hat{x}_{S}-\alpha\right)\left( \hat{\phi}+2\alpha\right)$ $=-\left\{ \left( 1+\frac{\alpha}{\beta_{G}x_{C}} \right)\frac{\alpha}{\beta_{G}x_{C}}p_{CC} \right\}\beta_{G}\beta_{L}\left( 1-G^{'} \right)\left( G^{'}\theta+1-\theta\right)+\alpha\left( 1-G^{'} \right)\left( \beta_{G}x_{C}+\alpha\right)$ $=\alpha\left( 1-G^{'} \right)\left( \beta_{G}x_{C}+\alpha\right)\left\{ 1-\frac{\beta_{L}}{\beta_{G}}\left( 1-\theta+G^{'}\theta\right)\frac{p_{CC}}{x_{C}^{2}} \right\}.$ |  |
| --- | --- | --- |

Then, the condition for $a_{3}<0$ is obtained, which is shown in inequality (8).

**Reference**

1. Diekmann O, Heesterbeek JAP, Metz JAJ. 1990 On the definition and the computation of the basic reproduction ratio R0 in models for infectious diseases in heterogeneous populations. *J. Math. Biol.* **28**, 365–382. (doi:10.1007/BF00178324)
